# Reconstructing the Holey Temple: A Composite Approach

**DOI:** 10.7759/cureus.22621

**Published:** 2022-02-26

**Authors:** Emma Russell, Jeremy Udkoff, Thomas Knackstedt, Aton Holzer

**Affiliations:** 1 School of Medicine, Case Western Reserve University School of Medicine, Cleveland, USA; 2 Department of Dermatology, University of Pittsburgh Medical Center, Pittsburgh, USA; 3 Department of Dermatology, Case Western Reserve University School of Medicine and MetroHealth System, Cleveland, USA; 4 Department of Dermatology, Tel Aviv Sourasky Medical Center, Tel Aviv, ISR

**Keywords:** dermatology and dermatologic surgery, skin graft, complex facial reconstruction, skin closure, mohs surgery

## Abstract

Surgical defects involving multiple facial cosmetic subunits can be challenging to reconstruct. We report on a patient with a complex temporal defect following Mohs micrographic surgery (MMS) for a basal cell carcinoma (BCC). The extension of the defect across the left temple, cheek, and forehead hindered the utilization of linear closures or flaps. Healing by secondary intention was considered but was determined to be a suboptimal approach given the involvement of the convex cheek. A modified full-thickness skin graft (FTSG) with linear closures of the distal poles of the wound was ultimately utilized, with excellent cosmetic results at three-month follow-up. Herein, the authors summarize this case and the indications for FTSG and secondary intention healing (SIH) for surgical defects involving the face.

## Introduction

Surgical defects spanning multiple cosmetic subunits can be challenging to reconstruct and may require multiple repair techniques to achieve a good cosmetic result. Secondary intention healing (SIH) or full-thickness skin grafts (FTSG) may be considered for complex defects not amenable to linear closures.

Healing by secondary intention can offer excellent results on the concave surface of the nose, around the eye, ear, and temple (NEET) areas of the face, where centripetal forces from scar contraction help minimize the scar appearance [1,2]. On the contrary, SIH for defects located on convex surfaces of the nose, oral lips, cheeks, chin, and helix of ear (NOCH), produce variable and often suboptimal results. Alternatively, FTSG can be useful in areas where linear closures or flaps may cause significant distortion, such as the periorbital area, ear, and nose [3]. However, FTSGs require a well-vascularized bed for the graft to survive, and therefore, may not always be a viable option based on the extent and location of the defect.

In this report, we present a case of a reconstruction using modified FTSG and linear closures in a patient with a complex temporal defect following Mohs micrographic surgery (MMS).

## Case presentation

**Figure 1:**
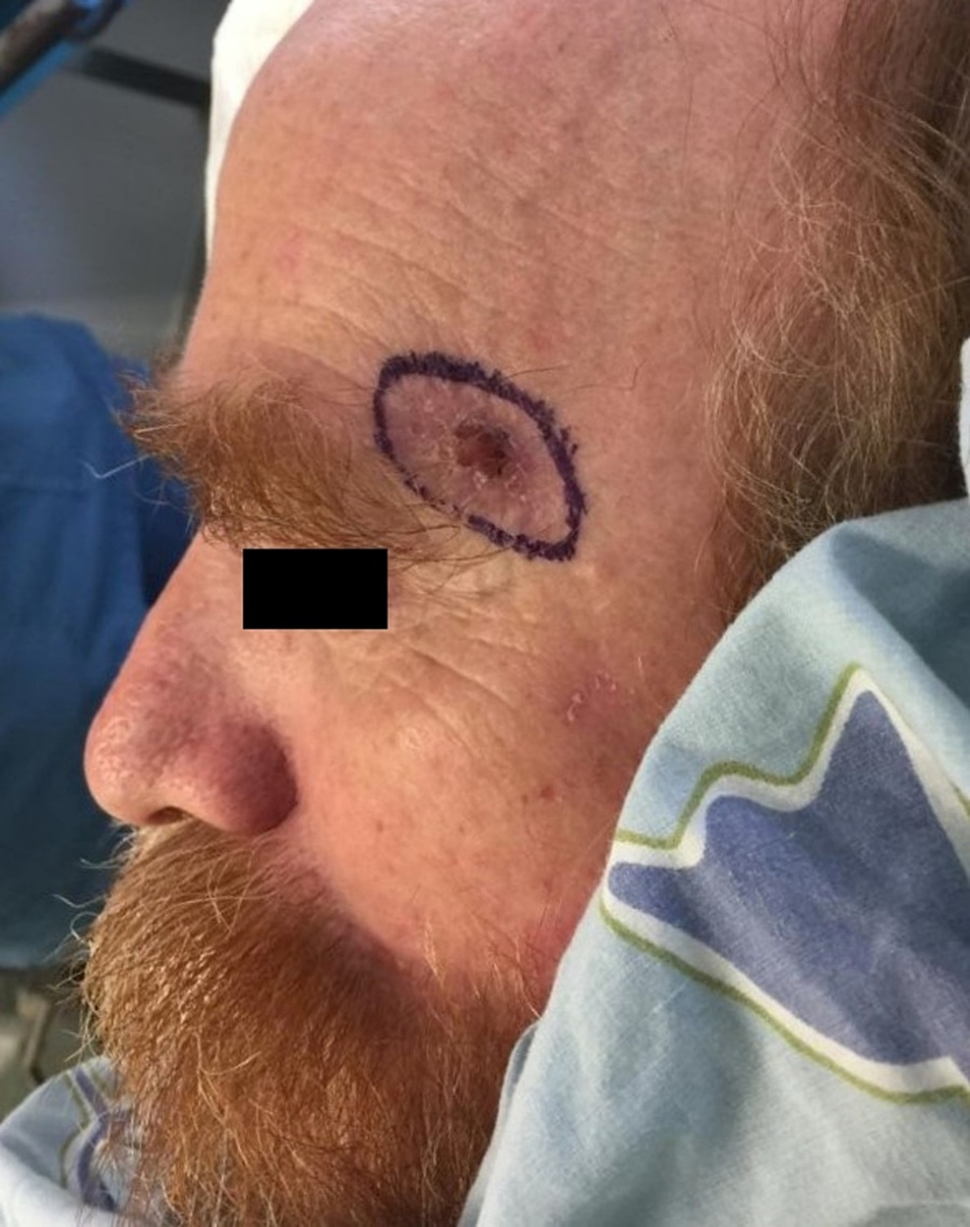
Basal cell carcinoma on the left lateral suprabrow

A 63-year-old man was referred for MMS for an infiltrative basal cell carcinoma (BCC) of the left lateral suprabrow. The tumor had undergone previous excision with subsequent linearly oriented recurrence along the wound edges (Figure 1).

**Figure 2:**
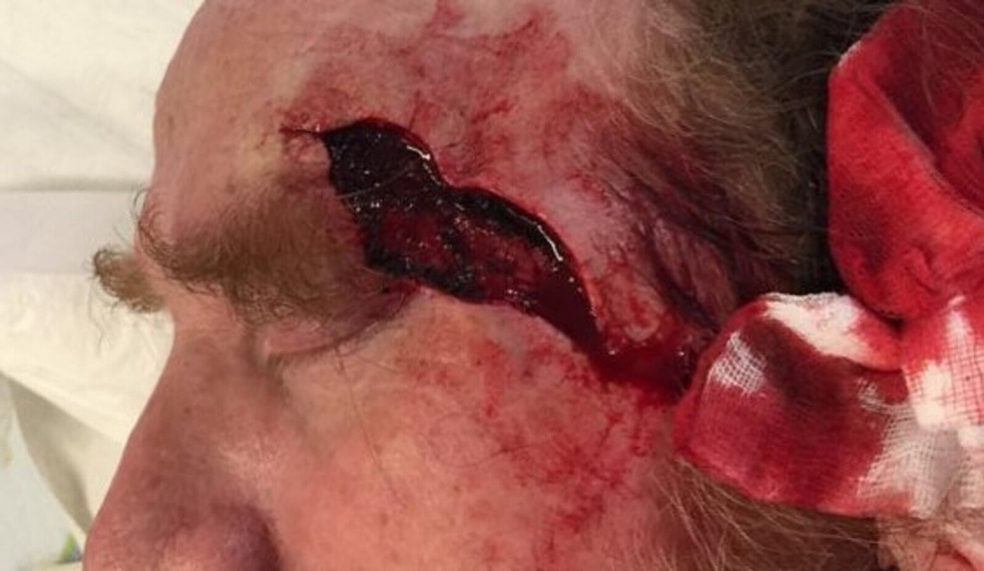
Defect following four Mohs micrographic surgery stages (8 x 3 centimeters)

The recurrence had much subclinical spread with indistinct margins and required four MMS stages for complete extirpation. The final defect size was 8 x 3 cm and spanned three cosmetic subunits across the left lateral forehead, temple, and cheek (Figure 2).

**Figure 3:**
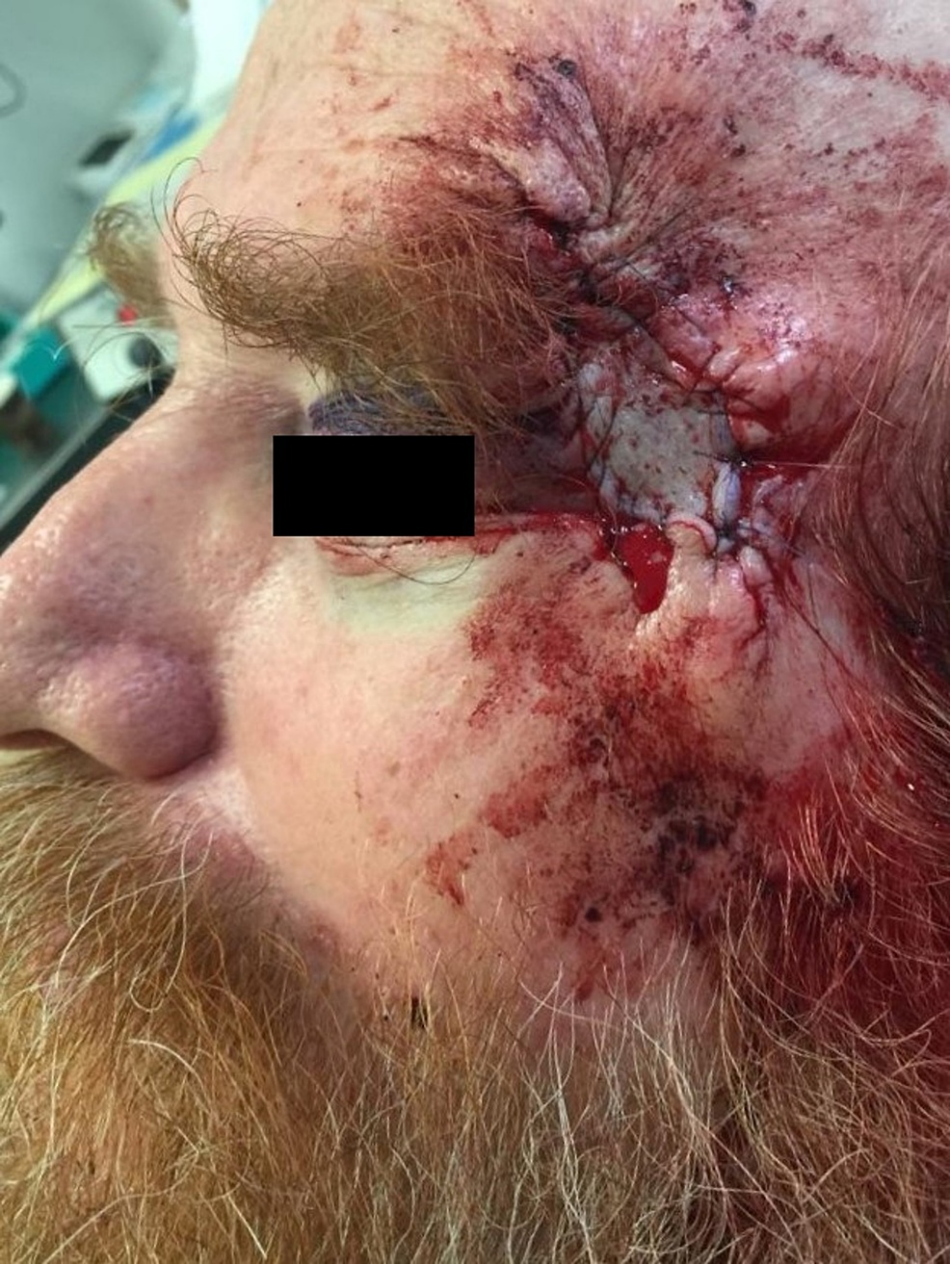
Linear closures on the distal poles of the wound and a full-thickness skin graft in the center

The wound was reconstructed under local anesthesia with 1% lidocaine with 1:100,000 epinephrine in the usual fashion. Undermining was performed out to one-half the width of the wound on the two distal ends. These areas were then approximated with linear closures. An approximately 4.5 x 3 cm defect remained at the center of the wound over the temple. A recipient template was created by placing 4 x 4 gauze on the remaining defect. This was then used to harvest a slightly larger modified FTSG (to accommodate for contraction) from post-auricular skin on the patient’s left side. This donor site was chosen primarily due to its proximity and close match to the color, texture, and thickness of the recipient site. An intradermal incision was made tracing the outline of the graft and a horizontally-oriented shave technique was then used to harvest a graft with a depth predominantly to the reticular dermis with some subcutaneous fat. The edges of the graft site were then approximated with a linear closure in the usual fashion. Any harvested fat was removed from the graft using curved Iris scissors. The graft was then secured at the recipient site with simple interrupted sutures (Figure 3).

Primary closure was used for the donor site. A pressure bandage was applied to the site and the patient was instructed to keep it on for 48 hours and then continue usual wound care.

**Figure 4:**
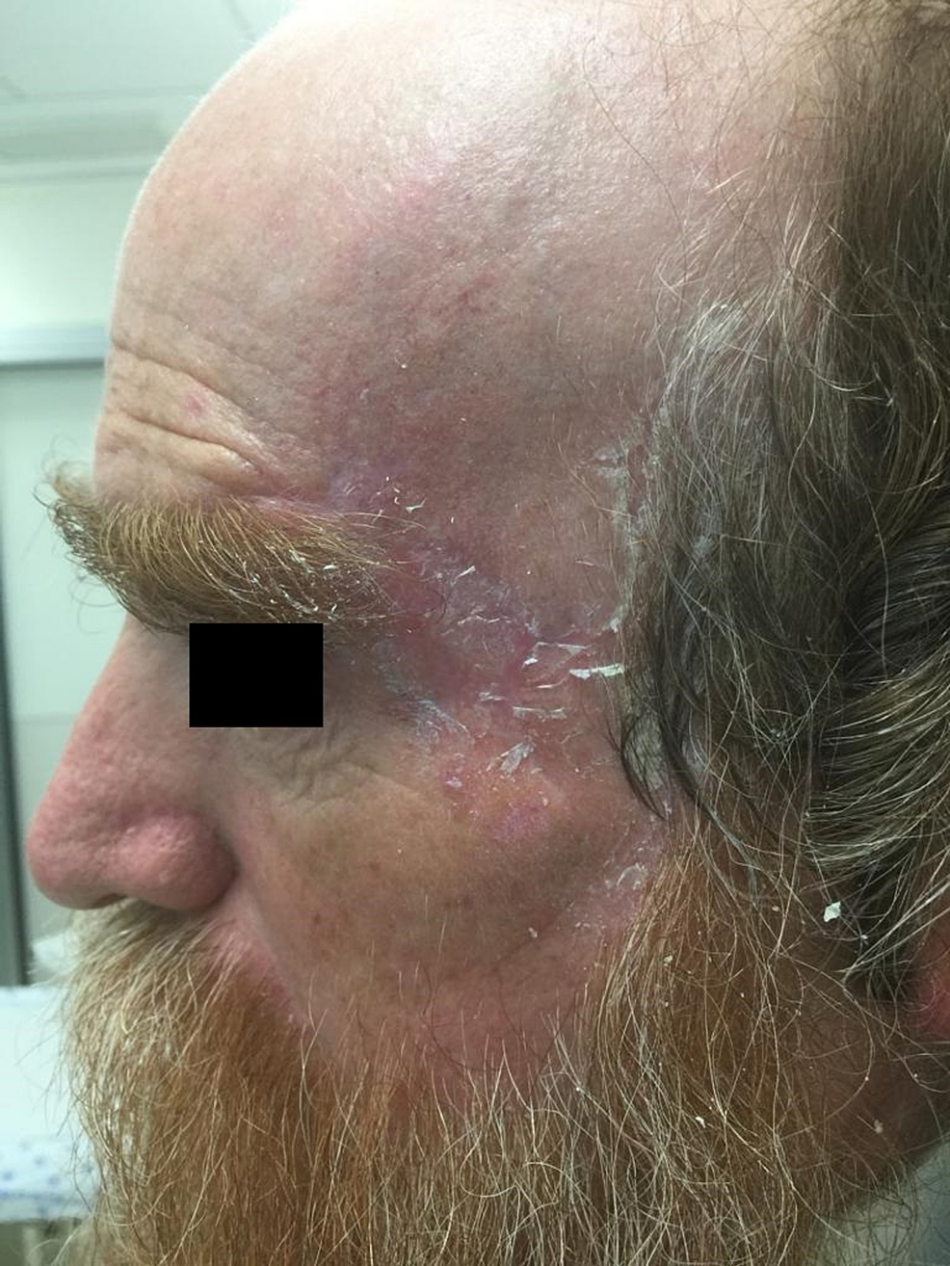
Three-month follow up visit with a good cosmetic outcome

At three months postoperatively, the wound and graft were well healed with good cosmetic appearance (Figure 4). There were no complications during the postoperative period. 

## Discussion

Surgical defects of the temple are typically repaired easily with horizontally oriented linear closures or flaps that mobilize the relatively lax surface tissues of the lower temple and cheek [4]. This option was not available for this particular defect, which encompassed the adjacent lower temple and cheek. In addition, even the adjacent forehead, which typically is a poor tissue reservoir as it is more tightly bound, was not available as the defect extended to this area as well.

SIH was considered, as the temple itself belongs to the NEET group of regions of the face that tend to yield acceptable cosmetic results with SIH, even if the temple itself is aided by less skeletal muscle contraction than other skin convexities [5]. However, the lower half of the defect overlaid the convex cheek which is a NOCH area and would be an unfavorable area for SIH. In the authors’ experience, SIH on the temple is more often complicated by exuberant granulation tissue. In addition, as with all SIH, hypopigmentation, and telangiectasis of the resultant scar is unpredictable [6].

A composite approach using an FTSG and linear closures for the edges was ultimately chosen due to the above constraints. The postauricular donor site proved to be a close match for the skin color, texture, and thickness of the involved cosmetic subunits, and, combined with the linear closures at the distal poles of the wound, the graft minimized any distortion of the involved area.

## Conclusions

This defect presented a challenge due to its involvement of numerous cosmetic subunits, including the left temple, cheek, and forehead. Its extension across these subunits and large size limited the utilization of linear closures or a flap. In addition, the varying convexities and concavities of the involved units made healing by secondary intention a suboptimal approach. Thus, an FTSG with linear closure of the distal poles of the wound was utilized. This resulted in an excellent cosmetic outcome with good preservation of facial contours, skin color, and texture.
